# Enhanced Elevated-Temperature Strength and Creep Resistance of Dispersion-Strengthened Al-Mg-Si-Mn AA6082 Alloys through Modified Processing Route

**DOI:** 10.3390/ma14195489

**Published:** 2021-09-23

**Authors:** Jovid Rakhmonov, Kun Liu, Paul Rometsch, Nick Parson, X.-Grant Chen

**Affiliations:** 1 Department of Applied Science, University of Quebec at Chicoutimi, Saguenay, QC G7H 2B1, Canada; jovid.rakhmonov@gmail.com; 2 Arvida Research and Development Center, Rio Tinto Aluminum, Saguenay, QC G7S 4K8, Canada; Paul.Rometsch@riotinto.com (P.R.); nick.parson@riotinto.com (N.P.)

**Keywords:** Al-Mg-Si-Mn alloy, extrusion, α-Al(MnFe)Si dispersoids, microstructure, mechanical properties, creep resistance

## Abstract

In the present work, we investigated the possibility of introducing fine and densely distributed α-Al(MnFe)Si dispersoids into the microstructure of extruded Al-Mg-Si-Mn AA6082 alloys containing 0.5 and 1 wt % Mn through tailoring the processing route as well as their effects on room- and elevated-temperature strength and creep resistance. The results show that the fine dispersoids formed during low-temperature homogenization experienced less coarsening when subsequently extruded at 350 °C than when subjected to a more typical high-temperature extrusion at 500 °C. After aging, a significant strengthening effect was produced by β″ precipitates in all conditions studied. Fine dispersoids offered complimentary strengthening, further enhancing the room-temperature compressive yield strength by up to 72–77 MPa (≈28%) relative to the alloy with coarse dispersoids. During thermal exposure at 300 °C for 100 h, β″ precipitates transformed into undesirable β-Mg_2_Si, while thermally stable dispersoids provided the predominant elevated-temperature strengthening effect. Compared to the base case with coarse dispersoids, fine and densely distributed dispersoids with the new processing route more than doubled the yield strength at 300 °C. In addition, finer dispersoids obtained by extrusion at 350 °C improved the yield strength at 300 °C by 17% compared to that at 500 °C. The creep resistance at 300 °C was greatly improved by an order of magnitude from the coarse dispersoid condition to one containing fine and densely distributed dispersoids, highlighting the high efficacy of the new processing route in enhancing the elevated-temperature properties of extruded Al-Mg-Si-Mn alloys.

## 1. Introduction

Al-Mg-Si-Mn 6xxx alloys have increasingly become the materials of choice for various construction and transportation applications requiring medium to high strength [1,2]. In these alloys, Mg and Si are mainly responsible for the precipitation of MgSi precipitates, which confer high strength. By contrast, Mn provides Zener pinning to retard the recrystallization of α-Al grains during subsequent processing through dispersoid formation during homogenization [2,3]. Consequently, deformed grains can impart high strength and impact toughness [4].

In Al-Mg-Si-Mn 6xxx alloys, the typical dispersoids formed during homogenization are α-Al(FeMn)Si, appearing with either simple cubic or body-centered cubic structures [5,6]. These dispersoids tend to form during heating and become relatively coarse (≈200 nm in equivalent diameter) during the soaking stage of industrial homogenization treatments typically conducted above 540 °C [5,7,8]. Recent studies have focused on the nucleation and growth of α-Al(FeMn)Si dispersoids to reveal new possibilities for increasing the number density of dispersoids while ensuring that they remain reasonably small (≈40 nm) [7,9]. These research efforts are generally driven by the relatively high maximum solubility of Mn in α-Al and its affordability compared to other slow-diffusing transition metals, such as Sc and Zr [10], which are used to enhance the high-temperature strength of Al alloys. The β′-MgSi precipitates formed during heating to the homogenization temperature in an Al-Mn-Mg alloy act as effective nuclei for α-Al(FeMn)Si dispersoids [11,12], thus substantially increasing their number density [13]. Moreover, a new low-temperature homogenization process was established to obtain a high number density of fine α-Al(FeMn)Si dispersoids while exhibiting enhanced Orowan strengthening and Zener drag effects [7,8,14].

Al-Mg-Si-Mn alloys are generally subjected to thermo-mechanical processing, such as extrusion, before reaching the final shape. Extrusion is a rapid process conducted typically above 500 °C, but the continuous generation of dislocations during extrusion results in ultrafast dynamic diffusion of solutes, which can induce the coarsening of dispersoids. Our recent study [8] has shown that dispersoids become very prone to coarsening during a typical extrusion process of 6082 alloy, and their average sizes almost double after extrusion compared with that after homogenization at a relatively low temperature (400 °C). Therefore, decreasing the extrusion temperature to below 400 °C in combination with low-temperature homogenization, at which both the diffusivity of Mn in Al and the coarsening of α-Al(FeMn)Si dispersoids are rather limited [13,15], might further retard the coarsening of dispersoids and provide a higher Orowan strengthening effect after the final processing stage.

Incorporating fine and densely distributed α-Al(FeMn)Si dispersoids into extruded Al-Mg-Si alloys might also enhance their elevated-temperature mechanical properties and creep resistance. In our previous work [8,14,16], the positive contribution of dispersoids to the yield strength (YS) at 300 °C was proven after extrusion. For instance, the YS at 300 °C for a 1% Mn 6082 alloy improved from 36 MPa for a dispersoid-free material to ≈ 60 MPa for one that was dispersoid-rich. Abdu et al. [17] studied the deformation behavior of AA6082 alloy during creep and concluded that the presence of Mg_2_Si particles led to higher creep resistance than aluminum without such particles owing to their effective interaction with the dislocations. Although β″/β′ precipitates are quickly transformed into the equilibrium coarse β-Mg_2_Si phase during thermal exposure at 300 °C [8], the high number of fine Al(FeMn)Si dispersoids is expected to be much more effective in enhancing the creep resistance of Al-Mg-Si-Mn alloys. However, no further studies regarding the influence of α-Al(FeMn)Si dispersoids on the strength and creep deformation behavior at elevated temperatures (250–350 °C) in 6xxx alloys have been reported in the literature, especially with relatively low homogenization and deformation temperatures.

In this study, we investigated the possibility of tailoring the processing route of extruded Al-Mg-Si-Mn AA6082 alloys to obtain fine and densely distributed α-Al(FeMn)Si dispersoids in the final microstructure, thereby enhancing the high-temperature strength and creep resistance. A modified extrusion process at a low temperature (350 °C) was introduced to study the evolution of the dispersoids during the extrusion process. In addition, the influence of homogenization and extrusion parameters on the elevated-temperature properties was evaluated and compared with that of a more conventional industrial extrusion process. A relationship between the microstructure, especially the dispersoid characteristics, and elevated-temperature properties was established.

## 2. Materials and Methods

### 2.1. Materials and Processes

Two Al-Mg-Si-Mn AA6082 alloys (Al-0.84Mg-1.02Si-0.23Fe-0.016Ti, in wt %) with different Mn contents of 0.5% and 1.0% were direct-chill (DC) cast. The 100 mm diameter DC cast billets were subjected to low-temperature homogenization at 400 °C for 5 h to promote the precipitation of α-Al(FeMn)Si dispersoids in the grain interiors. For comparison, the alloy containing 0.5% Mn was also homogenized at 550 °C for 5 h, which is more typical of a homogenization practice used in industry [1]. The billets were water-quenched after homogenization.

After homogenization, the billets were extruded into round bars with a diameter of 17.8 mm on the Rio Tinto 780-ton experimental press. Herein, a low extrusion temperature of 350 °C was compared with a conventional extrusion temperature of 500 °C. The billets were induction-heated to those temperatures, and the container temperature was matched to the billet temperature. Owing to the low extrusion temperature, a solution treatment at 540 °C for 10 min was applied after extrusion to dissolve Mg_2_Si intermetallics, which was followed by an aging treatment at 180 °C for 5 h, which was termed as “T6” in this study [18]. Both the homogenization treatment before extrusion and the solution as well as aging treatments after extrusion were conducted in a programmable electrical air circulating furnace (Pyradia Inc., Longueuil, QC, Canada) at a heating rate of 60 °C/h. The alloys and processing routes along with the alloy codes are listed in Table 1. For the alloy codes, 0.5Mn and 1Mn represent the Mn content. The first code after the Mn content indicates the homogenization temperature (“H” for 550 °C and “L” for 400 °C), and the second code denotes the extrusion temperature (“H” for 500 °C and “L” for 350 °C) used in the present study.

### 2.2. Material Characterization

Scanning electron microscopy (SEM, JEOL-6480LV, Tokyo, Japan) coupled with electron backscatter diffraction (EBSD) and transmission electron microscopy (TEM, JEOL JEM-2100, Tokyo, Japan) were used to observe the microstructures of the alloys at different process stages. The EBSD analysis was performed on samples prepared with the standard grinding and polishing procedures using a suspension of 0.05 µm colloidal silica on the final polishing step. The step sizes were set at 0.3–0.5 and 7 µm for the unrecrystallized and recrystallized grains, respectively. The EBSD runs for each condition were assured to have an indexing ratio of at least 85%. The original EBSD datasets were post-processed using HKL Channel 5 software (Oxford Instruments HKL, Hobro, Denmark), and the noise reduction was performed to remove the non-indexed points. Samples with much coarser grains after extrusion were electro-etched using Barker’s reagent (3 vol % HBF4 solution) at 15 V for 3 min and observed under optical microscopy with polarized light. The distribution of the precipitates and dispersoids was observed using TEM. The preparation methods of thin foils for TEM analysis (operated at 200 kV) are described in a previous study [8] in which TEM foils were prepared by twin-jet polishing of 3 mm-diameter specimen punched from a mechanically grinded and polished to ≈50 µm thickness disk at 20 V DC using a solution of 67% methanol and 33% HNO_3_ at ≈20–30 °C. The measurement of foil thickness in the viewing direction was based on the Kossel–Möllenstedt (K-M) fringes obtained in the convergent beam electron diffraction pattern under the two-beam conditions [19]. The methods used to measure the volume fraction and number density of α-Al(FeMn)Si dispersoids have been described in a previous study [8] and are summarized as follows:

The volume fraction of Al(FeMn)Si dispersoids, $\;f_{d}$, was estimated by Equation (1), and their number density was calculated by Equation (2):

(1) $$f_{d}=A_{d}\frac{\bar{K}\bar{D}}{\bar{K}\bar{D}+t}\left(1-A_{DFZ}\right)$$

(2) $$N_{v}=\frac{N}{A\left(\bar{D}+t\right)}$$

where $\bar{D}$ is the dispersoid’s equivalent diameter; *t* is the TEM foil thickness, $A_{d}$ is the percentage area of dispersoids from the TEM images, $A_{DFZ}$ is the percentage area of dispersoid-free zones from the OM images; $\bar{K}$ is the average shape factor for dispersoids, and the value of 0.45 is used in this study. $N_{v}$ represents the number of dispersoids within the image, and *A* is the area of the image. At least six TEM micrographs captured from different regions of the α-Al matrix were used to quantify the dispersoids.

Compression tests were performed using a Gleeble 3800 thermo-mechanical simulator (Dynamic Systems Inc., Austin, TX, USA) unit on cylindrical specimens with a length of 15 mm and a diameter of 10 mm to determine the compressive YS. The strain rate was set at 10^−3^ s^−1^. The YS was measured at 20 °C after the T6 aging treatment and at both 20 and 300 °C after thermal exposure for 100 h at 300 °C following the aging treatment. Compression tests at 300 °C involved preheating of the specimen to the test temperature (2 °C/s heating rate), holding the specimen at the test temperature for 180 s, and then compressing the specimen to a strain of 0.2. The temperature of the specimen during testing was maintained at 300 ± 0.2 °C. Repeatability was ensured by testing four samples per condition. The creep tests were conducted in compression mode at 300 °C with the same specimen dimensions as in the Gleeble tests. For each creep test, a constant load was applied to provide initially applied stress varying from 20 to 40 MPa for the duration of 100 h.

## 3. Results

### 3.1. As-Extruded and Solutionized Microstructures

**Figure 1:**
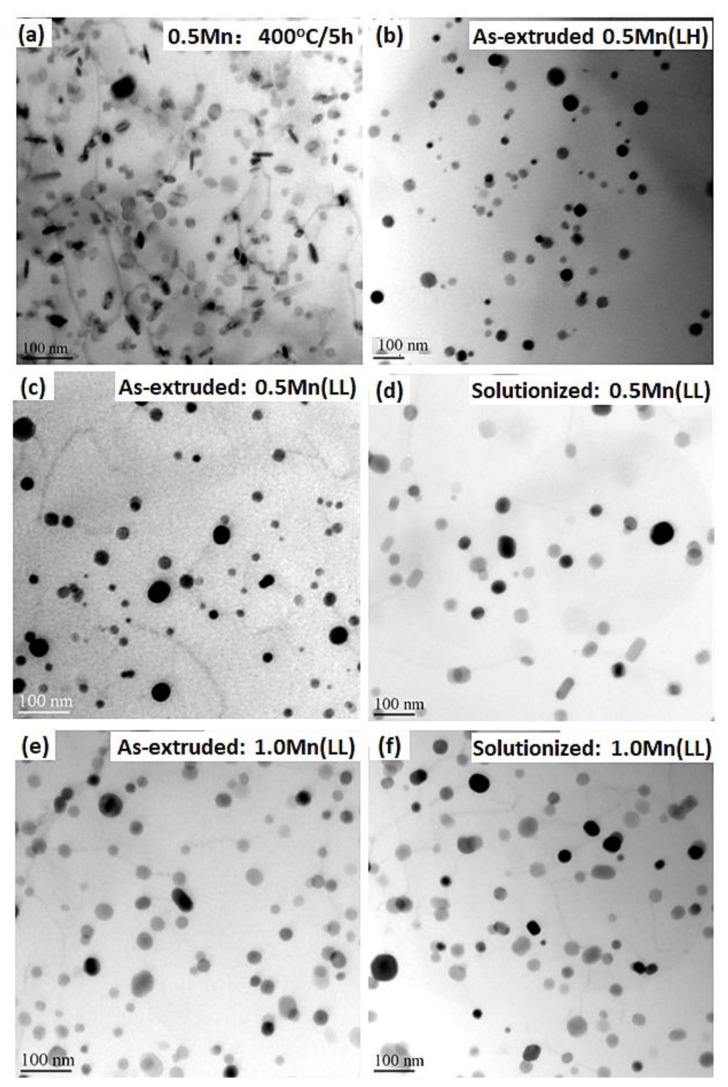
Distribution of α-Al(FeMn)Si dispersoids under various conditions: (**a**) 0.5Mn homogenized at 400 °C/5 h; (**b**) as-extruded 0.5Mn(LH); (**c**) as-extruded 0.5Mn(LL); (**d**) solutionized 0.5Mn(LL); (**e**) as-extruded 1Mn(LL) and (**f**) solutionized 1Mn(LL).

Figure 1 shows the evolution of the dispersoids under various conditions in both the 0.5 Mn and 1 Mn alloys. For the 0.5 Mn alloy homogenized at 400 °C for 5 h (Figure 1a), numerous fine α-Al(FeMn)Si dispersoids (cubic with *a* = 1.265 nm [6]) with an average equivalent diameter ($\bar{D}$) of ≈25 nm precipitated in the Al matrix. During the conventional extrusion at 500 °C (Figure 1b), the fine dispersoids obtained after homogenization experienced substantial coarsening, as their $\bar{D}$ increased from ≈25 nm after homogenization at 400 °C/5 h to ≈40 nm after extrusion (0.5Mn(LH)).

However, under modified extrusion at 350 °C (Figure 1c), the dispersoids exhibited no appreciable coarsening, and they maintained a fine size ($\bar{D}$ of ≈25 nm, 0.5Mn(LL) in Table 2). With a short-time solution treatment (540 °C/10 min, Figure 1d) after extrusion, the dispersoids underwent moderate coarsening compared with the 350 °C as-extruded condition. As shown in Table 2, the size of the dispersoids after solutionizing slightly increased (33 nm vs. 25 nm) with reduced number density ($N_{v}$, 387 µm^−3^ vs. 859 µm^−3^) compared with the as-extruded condition. Despite their coarsening after solutionizing, the dispersoids in 0.5Mn(LL) (Figure 1d) were still finer than those extruded at 500 °C (0.5Mn(LH), Figure 1b). This can most likely be attributed to the fact that the continuous generation of dislocations during high-temperature extrusion provides a much higher rate of solute diffusion [15], causing faster coarsening of dispersoids compared to the solutionized condition.

A similar tendency was also observed in the 1 Mn alloy in which the dispersoids were fine with a high number density in the as-extruded condition (Figure 1e) and underwent a moderate coarsening after the short-time solution treatment (Figure 1f). Furthermore, the number density ($N_{v}$) in 1Mn(LL) after solutionizing was remarkably higher than that in 0.5Mn(LL) (588 µm^−3^ vs. 387 µm^−3^) with a comparable size of ≈33 nm (Table 2), resulting from the higher Mn content in 1Mn(LL).

By contrast, homogenization at a higher temperature before extrusion negatively impacts the dispersoid size and number density [8,13,16]. For instance, although extruded at 350 °C, the dispersoids formed during high-temperature homogenization (550 °C/5 h) in 0.5Mn(HL) featured a much larger $\bar{D}$ and lower $N_{v}$ after extrusion compared to the dispersoids formed during low-temperature homogenization (400 °C/5 h) in 0.5Mn(LL), as shown in Table 2.

**Figure 2:**
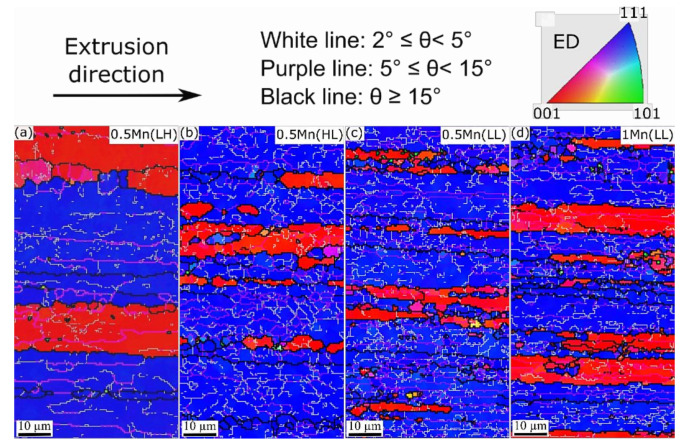
EBSD inverse pole figure (IPF) maps of as-extruded (**a**) 0.5Mn(LH), (**b**) 0.5Mn(HL), (**c**) 0.5Mn(LL), and (**d**) 1Mn(LL), showing the grain and subgrain structures at the center of the extruded rods, and the crystallographic orientations of grains along the ED.

**Figure 3:**
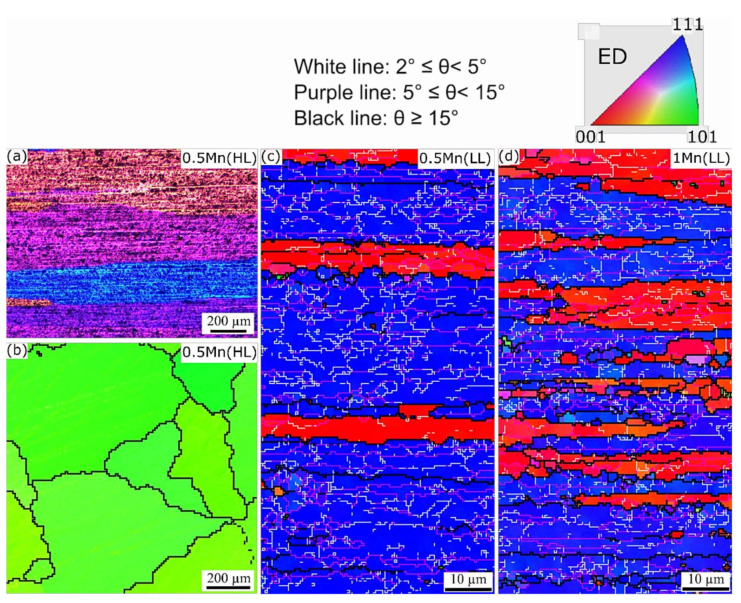
(**a**) Deformed grains from 0.5 Mn (HL) under optical microscopy and (**b**–**d**) the IPF maps acquired from solutionized alloys: (**b**) 0.5Mn(HL), (**c**) 0.5Mn(LL), and (**d**) 1Mn(LL), showing the grain/subgrain structures and the crystallographic orientations. Maps were obtained from the surface parallel (**a**,**c**,**d**) and perpendicular (**b**) to the extrusion direction.

EBSD scans were performed to compare the grain structures in the as-extruded and solutionized states, and the obtained inverse pole figure (IPF) maps are displayed in Figure 2 and Figure 3. In the as-extruded state (Figure 2), all the samples exhibited fibrous grains elongated along the extrusion direction (ED). Typical double <001>Al//ED and <111>Al//ED axisymmetric deformation texture components were observed under all conditions. The presence of low-angle grain boundaries (LAGBs), particularly those with misorientations between 5° and 15°, indicates that dynamic recovery occurred during extrusion, resulting in the formation of a combination of equiaxed and elongated subgrains. Misorientations lower than 5° in the IPF maps suggest that the dynamic recovery was partial. From IPF maps in Figure 2, it is found that the alloys extruded at 350 °C possessed the higher amount of stored energy in the form of dislocations and the higher quantity of subgrain boundaries (Figure 2b–d) compared to that extruded at 500 °C in 0.5Mn(LH) (Figure 2a).

Upon solutionizing (Figure 3), 0.5Mn(HL) underwent static recrystallization (SRX), resulting in the formation of very coarse grains (2–4 mm in length) that are still elongated along the ED (Figure 3a). This same sample exhibited a strong <011>Al//ED texture component (Figure 3b), which replaced the extruded <001>Al//ED and <111>Al//ED deformation texture components (Figure 2a). Apparently, the very coarse dispersoids in 0.5Mn(HL) (Table 2) were incapable of retarding SRX. By contrast, both 0.5Mn(LL) and 1Mn(LL) remained resistant to SRX at 540 °C, and the microstructures under both conditions were still deformed recovery structures (Figure 3c,d) owing to the higher Zener pinning effect caused by the fine and densely distributed dispersoids [13,16]. In addition, compared with the grain structure in 0.5Mn(LH) (Figure 2a), the 0.5Mn(LL) after solution treatment (Figure 3c) still had a higher density of dislocations and LAGBs. This likely resulted from the finer size and higher number density of dispersoids in 0.5Mn(LL) (Figure 1d), indicating the positive influence of dispersoids on the static recrystallization resistance resulting from the tailored thermo-mechanical process in this study.

### 3.2. Room-Temperature Mechanical Properties and Microstructure after T6/T5 Treatment

**Figure 4:**
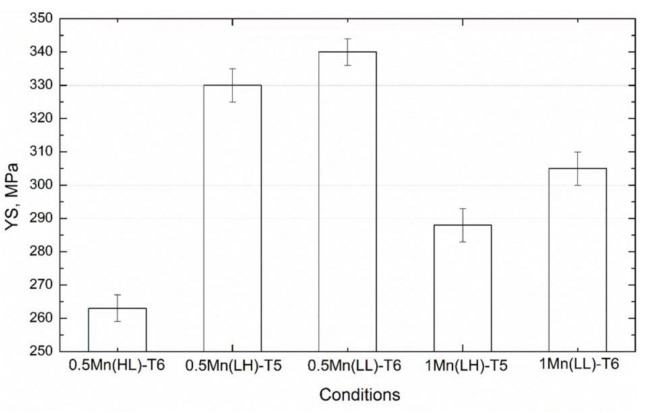
Room-temperature YS of the experimental alloys after aging treatment.

**Figure 5:**
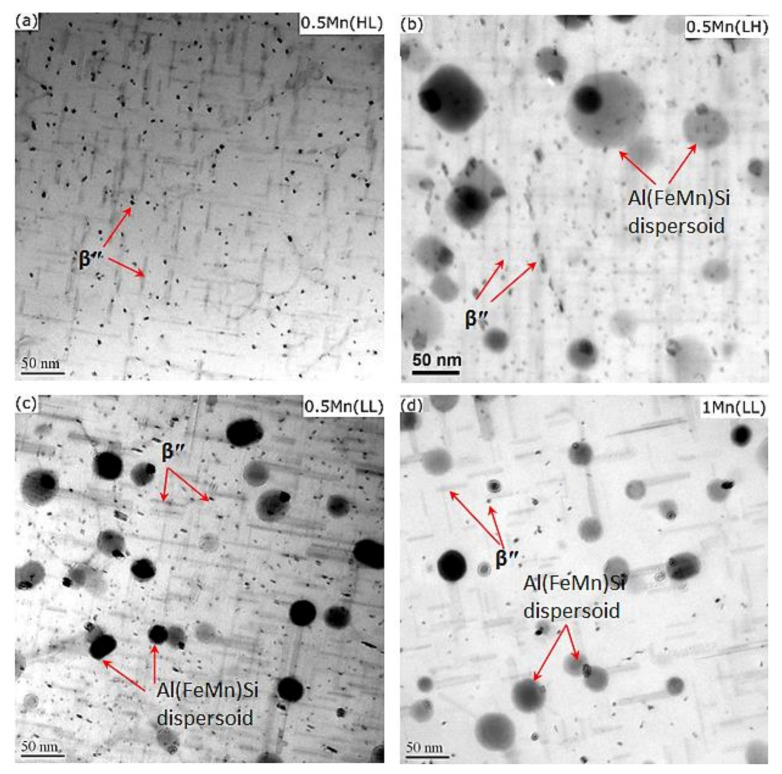
Representative bright-field TEM images showing β″ precipitates and α-Al(FeMn)Si dispersoids in (**a**) 0.5Mn(HL), (**b**) 0.5Mn(LH), (**c**) 0.5Mn(LL), and (**d**) 1Mn(LL).

Figure 4 displays the YS at room temperature (RT) of the experimental alloys after the T6/T5 treatments. The YS was the lowest in 0.5Mn(HL) (262 MPa), which was homogenized at 550 °C for 5 h, while the YS significantly increased to 340 MPa in 0.5Mn(LL) and then moderately decreased to 305 MPa in 1Mn(LL). These trends can be attributed to the different precipitation behaviors in the experimental alloys during the T6 treatment, as shown in Figure 5.

In the T6 condition, a large number of needle-shaped precipitates were present in the aluminum matrix. These are identified as β″-MgSi (monoclinic with *a* = 1.516 nm, *b* = 0.405 nm, *c* = 0.674 nm, and β = 105.3° [20]) according to their morphology and size [20]. In 0.5Mn(HL) (Figure 5a), numerous β″ precipitates provided most of the strengthening of the aluminum matrix [21]. However, the dispersoids were difficult to find and hence had a negligible effect on the strengthening owing to their large size and very low number density (Table 2). The microstructures of 0.5Mn(LL) and 1Mn(LL) were quite distinct from those of 0.5Mn(HL). In both alloys (Figure 5c,d), not only abundant β″ precipitates but also a high number of dispersoids co-existed in the aluminum matrix. The dispersoids together with the highly deformed grain structures (Figure 3) provided a complementary strengthening effect in addition to β″ strengthening; hence, a remarkably higher YS was achieved in 0.5Mn(LL) and 1Mn(LL) compared to 0.5Mn(HL). Upon closer observation, it is evident that 1Mn(LL) had a lower number density of β″ and a larger interparticle spacing between β″ precipitates (Figure 5d) than 0.5Mn(LL) (Figure 5c), resulting in a lower YS relative to 0.5Mn(LL). This is most likely because the higher Mn alloy (1Mn(LL)) has more Si tied up in the Fe-rich intermetallics and α-Al(FeMn)Si dispersoids, thereby leaving less Si for β″ formation during aging.

For comparison with the conventional extrusion at high temperature (500 °C), the YS of both 0.5Mn and 1Mn alloys under T5 from our previous work [8] are also included in Figure 4, labeled 0.5Mn(LH) and 1Mn(LH). YS of the two alloys under different extrusion conditions is comparable. The 0.5Mn(LL) and 1Mn(LL) samples extruded at low temperature (350 °C) exhibited higher YS than their counterparts (0.5Mn(LH) and 1Mn(LH)). The microstructure of 0.5Mn(LH) (Figure 5b) was similar to that of 0.5Mn(LL); β″ precipitates and dispersoids co-existed in the matrix (Figure 5b,c). However, the dispersoids in 0.5Mn(LH) were coarser with a lower number density compared to those in 0.5Mn(LL) (Table 3) because of the faster coarsening during high-temperature extrusion. The finer dispersoids and more severely deformed grain structures in 0.5Mn(LL) and 1Mn(LL) are most likely responsible for their slightly higher YS when compared to 0.5Mn(LH) and 1Mn(LH).

Regarding the effect of homogenization temperature, it is clear that the fine and densely distributed dispersoids formed during low-temperature homogenization, regardless of the extrusion route, remarkably enhanced the YS. For instance, in the 0.5 Mn alloy, 0.5Mn(LL) and 0.5Mn(LH) significantly improved the YS in the T5/T6 states by 72–77 MPa (≈28%) relative to 0.5Mn(HL), which had a very low number density of dispersoids and coarse recrystallized grains.

### 3.3. Elevated-Temperature Strength and Microstructure after Thermal Exposure at 300 °C/100 h

**Figure 6:**
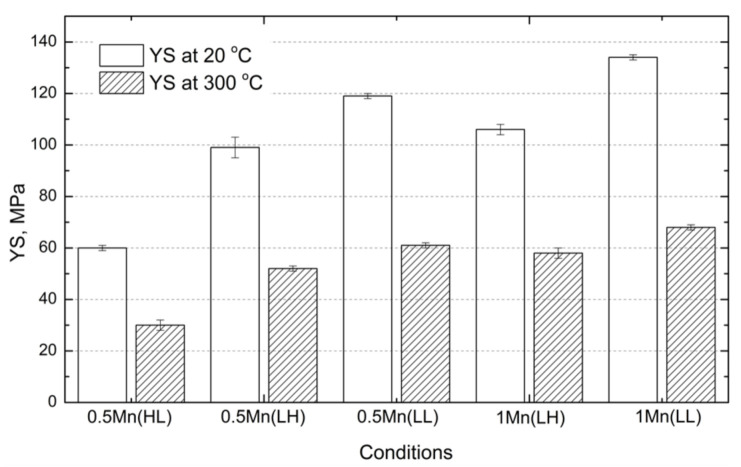
Room- and elevated-temperature YS of experimental alloys after thermal exposure at 300 °C/100 h.

To explore the possibility of using Al-Mg-Si-Mn alloys in elevated-temperature applications and to reveal the effect of fine dispersoids on the elevated-temperature strength, the alloys were further conditioned at 300 °C for 100 h to stabilize the microstructure. Then, compressive tests were conducted to evaluate the mechanical properties at both 20 and 300 °C, and the results are shown in Figure 6. For comparison purposes, the results of 0.5Mn(LH) and 1Mn(LH) under the same thermal exposure conditions as in our previous work [8] are also included in Figure 6.

Compared with the YS after aging (Figure 4), the YS after thermal exposure at all conditions was much lower. For instance, the YS at 20 °C for 0.5Mn(LL) after aging reached 340 MPa, but it sharply decreased to 119 MPa after thermal exposure. This is a typical overaging phenomenon, which is a significant challenge for such alloys in elevated-temperature applications. This is principally due to the complete transformation of fine strengthening precipitates of β″ into coarse β-Mg_2_Si particles during thermal exposure, which have negligible strengthening effects in the aluminum matrix.

For the strength after thermal exposure among the experimental alloys, a similar tendency was observed at both 20 and 300 °C; the lowest strength was obtained in 0.5Mn(HL) as expected, and then it increased from 0.5Mn(LL) to 1Mn(LL). As an example, the YS at 300 °C after thermal exposure increased from 30 MPa for 0.5Mn(HL) to 61 MPa for 0.5Mn(LL) and further to 68 MPa for 1Mn(LL), which is more than a doubling of the elevated-temperature strength. On the other hand, compared with the conventional extrusion at high temperature, the alloys subjected to modified low-temperature extrusion exhibited higher YS at both 20 °C and 300 °C. For instance, in both 0.5Mn and 1Mn alloys, there was ≈17% improvement in the YS at 300 °C (61 MPa for 0.5Mn(LL) vs. 52 MPa for 0.5Mn(LH) and 68 MPa for 1Mn(LL) vs. 58 MPa for 1Mn(LH)). Even the YS at 300 °C for 0.5Mn(LL) was higher than that for 1Mn(LH) extruded at 500 °C (61 MPa vs. 58 MPa).

**Figure 7:**
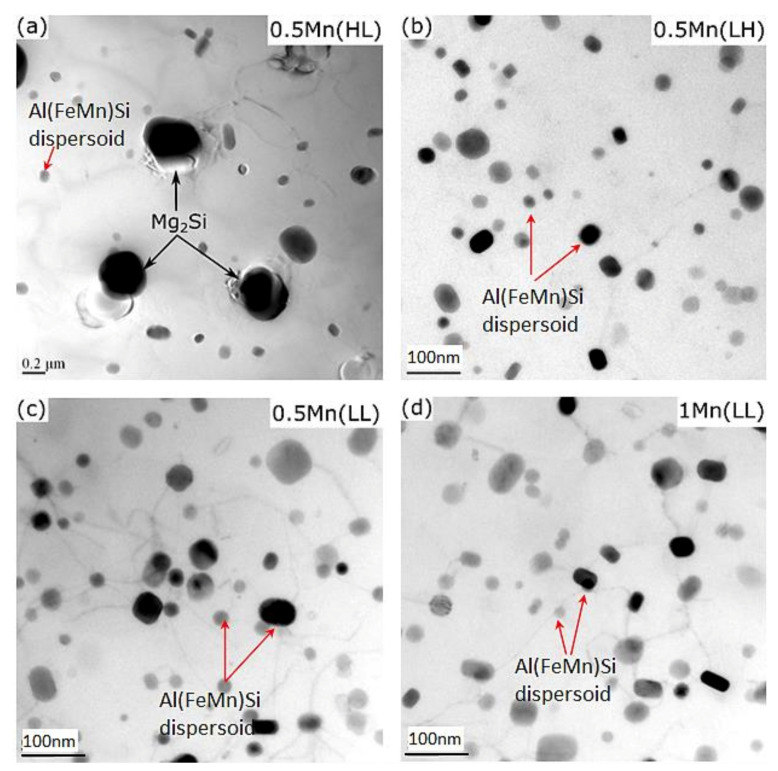
Bright-field TEM images showing Al(FeMn)Si dispersoids embedded in the Al matrix of the experimental alloys after thermal exposure at 300 °C for 100 h in (**a**) 0.5Mn(HL), (**b**) 0.5Mn(LH), (**c**) 0.5Mn(LL), and (**d**) 1Mn(LL).

The distributions of the precipitates and dispersoids after thermal exposure are shown in Figure 7. As shown in Figure 7a, the β″ precipitates were completely transformed into equilibrium β-Mg_2_Si particles (cubic with *a* = 0.635 nm [20]) after thermal exposure, resulting in a complete loss of a strengthening effect. By contrast, the Al(FeMn)Si dispersoids exhibited no appreciable coarsening during thermal exposure. Hence, only dispersoids that were thermally stable at 300 °C remained the predominant strengthening phases in the experimental alloys. The morphology and distribution of the dispersoids are shown in Figure 7b–d for 0.5Mn(LH), 0.5Mn(LL), and 1Mn(LL), respectively. As shown in Figure 7a, the dispersoids co-existed with a few coarse β-Mg_2_Si particles in 0.5Mn(HL). However, the dispersoids in 0.5Mn(HL) were large, and the number density was low, leading to the lowest YS. By contrast, the dispersoids became much finer with increasing number density from 0.5Mn(LL) to 1Mn(LL) (Table 3), leading to a higher YS in 0.5Mn(LL) and a further improvement in 1Mn(LL). It is worth mentioning that the fine dispersoids can be more effectively retained under modified low-temperature extrusion conditions compared with conventional extrusion parameters. As shown in Table 3, the dispersoids in 0.5Mn(LL) were finer with a higher number density compared with those in 0.5Mn(LH), and similarly for 1Mn(LL) vs. 1Mn(LH). Therefore, the YS values after thermal exposure were further enhanced by modified low-temperature extrusion.

For comparison purposes, the quantitative TEM results of the dispersoids and mechanical properties under all conditions are summarized in Table 3. Homogenization at a high temperature (550 °C/5h) generated a few coarse dispersoids (0.5Mn(HL)), resulting in the lowest mechanical properties after aging and after thermal exposure. Compared with conventional extrusion at high temperatures (0.5Mn(LL) and 1Mn(LL) vs. 0.5Mn(LH) and 1Mn(LH)), it can be concluded that the modified processing route at lower extrusion temperature results in finer and more densely distributed dispersoids in the final microstructures of Al-Mg-Si-Mn alloys. This provides a complementary strengthening effect on the room-temperature mechanical properties after aging in addition to the traditional β″ precipitate strengthening. After thermal exposure at 300 °C, the dispersoids became the predominant strengthening phase. The benefits of the finer and more densely distributed dispersoids obtained by the new processing route become even more obvious, providing both alloys (0.5Mn and 1Mn) with excellent YS at elevated temperatures and achieving a 17% improvement in the YS at 300 °C compared to the conventionally extruded ones.

### 3.4. Creep Deformation and the Associated Microstructure

**Figure 8:**
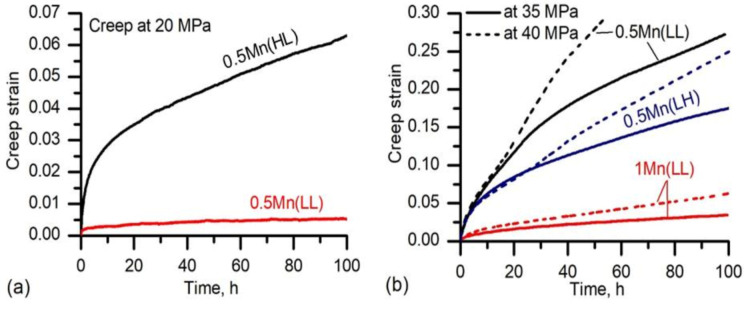
Typical creep curves from tests performed at 300 °C under the applied stress of (**a**) 20 MPa, (**b**) 35 MPa, and 40 MPa.

The creep properties, as one of the most significant properties at elevated temperatures [22,23], were further evaluated on the experimental alloys at 300 °C. Owing to the relatively low YS of 30 MPa at 300 °C for 0.5Mn(HL), the creep tests were first started at an applied stress of 20 MPa for 0.5Mn(HL) and 0.5Mn(LL). The resulting creep curves are shown in Figure 8a. The total creep strain after 100 h in 0.5Mn(LL) was almost ten-fold smaller than that in 0.5Mn(HL) (0.005 for 0.5Mn(LL) vs. 0.06 for 0.5Mn(HL)). Meanwhile, the minimum creep rate calculated from the quasi-steady-state region was 4.8 × 10^−9^ s^−1^ for 0.5Mn(LL), which was only a tenth of that in 0.5Mn(HL) (6.5 × 10^−8^ s^−1^). Therefore, superior creep resistance is retained for 0.5Mn(LL) over 0.5Mn(HL), which resulted from the much higher number density of finer dispersoids in 0.5Mn(LL) after thermal exposure than that in 0.5Mn(HL) (Table 3).

Furthermore, different creep stresses ranging from 20 to 40 MPa were applied to 0.5Mn(LL), 0.5Mn(LH), and 1Mn(LL) in the creep tests. Figure 8b displays their typical creep curves under two applied stresses of 35 and 40 MPa. 1Mn(LL) presented the lowest total creep strain and creep rate among the three conditions, showing the best creep resistance. Samples 0.5Mn(LH) and 0.5Mn(LL) displayed a higher total creep strain and creep rate, and hence a lower creep resistance compared with 1Mn(LL). Unexpectedly, the creep resistance of 0.5Mn(LL) was slightly lower than that of 0.5Mn(LH) at both 35 and 40 MPa. For instance, the total creep strain after 100 h at 35 MPa in 0.5Mn(LL) was 0.25 compared with 0.19 in 0.5Mn(LH).

**Figure 9:**
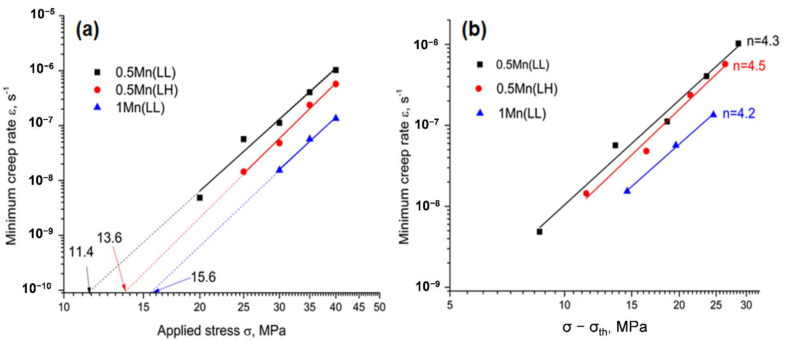
Determination of threshold stress *σ_th_* in the plots of the minimum creep strain rates vs. applied stresses (**a**) and the true stress exponent *n* in the plots of the minimum creep strain rates vs. the effective stresses (**b**).

To better understand the creep behavior of these experimental alloys, two important creep parameters (threshold stress (*σ_th_*) and true stress exponent (*n*)) were calculated. Figure 9a shows the minimum creep rate *ε* as a function of the applied stress on a logarithmic scale. The values of the threshold stress *σ_th_* were obtained when the minimum creep rates (*ε*) at different stresses were extrapolated by linear fitting to the creep rate of 10^−10^ s^−1^, below which the creep is experimentally not measurable [24,25]. As shown in Figure 9a, the minimum creep rate *ε* was always the lowest in 1Mn(LL) followed by 0.5Mn(LH) and 0.5Mn(LL) at all applied stresses, and the threshold stress *σ_th_* increased from 11.4 MPa in 0.5Mn(LL) to 13.6 MPa in 0.5Mn(LH) and further to 15.6 MPa in 1Mn(LL). It is evident that 1Mn(LL) is significantly more creep-resistant than 0.5Mn(LH) and 0.5Mn(LL).

After determining the threshold stress *σ_th_*, the creep behavior of the alloys can be generally described by a modified power-law equation [26,27] in which the true stress exponent n can be determined as follows:

(3) $$\varepsilon =A{\left(\sigma -\sigma_{th}\right)}^{n}\exp\left(-\frac{Q}{RT}\right)$$

where σ is the applied stress, *σ_th_* is the threshold stress, *σ−σ_th_* is the effective stress, *n* is the true stress exponent, *Q* is the activation energy, *A* is a dimensionless constant, *R* is the ideal gas constant, and T is the absolute temperature.

**Figure 10:**
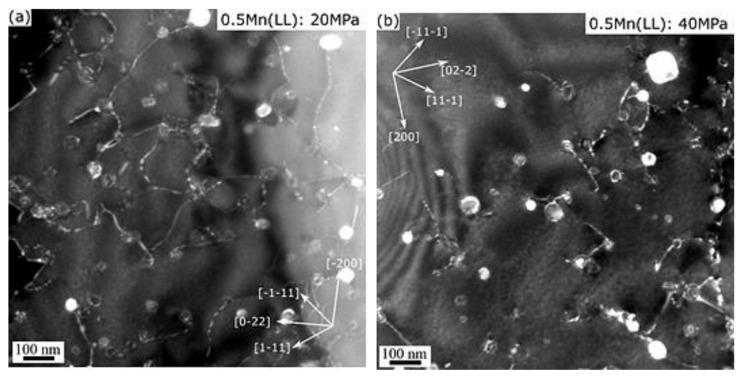
Weak-beam dark-field TEM images showing the interaction between dislocations and Al(FeMn)Si dispersoids in 0.5Mn(LL) creep samples under applied stresses of (**a**) 20 MPa and (**b**) 40 MPa.

Figure 9b shows the plots of the minimum creep rate *ε* against the effective stress *σ−σ_th_* on a logarithmic scale. The slopes of the ln*ε*−ln(*σ−σ_th_*) curves determined the values of the true stress exponent *n* [25]. In the literature, *n* is frequently used to identify the mechanisms that control creep deformation. The true stress exponent *n* was calculated to be between 4.2 and 4.5 for the three experimental conditions (Figure 9b), which suggests that creep in these samples is controlled by the dislocation climb mechanism [26,28]. Figure 10 shows the TEM analysis of the 0.5Mn(LL) samples, which revealed the presence of dislocations pinned by dispersoids during climbing, confirming the dislocation-climb-controlled creep mechanism. The interactions between the dispersoids and dislocations in the 0.5Mn(LL) samples under both 20 and 40 MPa are shown in Figure 10. It is apparent that the presence of dispersoids, which effectively interact with the dislocations, plays a significant role in the creep resistance. The coarse β resulting from the β″ transformation during the thermal exposure before creep contributed less to strengthening and creep resistance (Figure 7a). As shown in Table 3, the highest number density of fine dispersoids was retained in 1Mn(LL) among all the experimental alloys and conditions, which contributed to its high creep resistance.

Although 0.5Mn(LL) possessed more dispersoids with finer sizes compared to 0.5Mn(LH) before creep (Table 3), its creep resistance was found to be lower than that of 0.5Mn(LH) (Figure 8b and Figure 9), which might have resulted from the different grain structures under these two conditions. As shown in Figure 3c, the low-temperature extrusion (350 °C) in 0.5Mn(LL) yielded finer dispersoids but also introduced a higher amount of LAGBs (i.e., more subgrains and subgrain boundaries) than 0.5Mn(LH) extruded at 500 °C (Figure 2a). In addition to the positive effect of dispersoids on the creep resistance, the more highly deformed structure of 0.5Mn(LL) may play an opposite role because it can increase the climb rate of dislocations over dispersoids as the climbing process is vacancy-mediated [27]. The higher number of subgrains and their boundaries in 0.5Mn(LL) compared to 0.5Mn(LH) might act as additional vacancy sources at elevated temperatures, thus enhancing the dislocation climb during creep and reducing creep resistance.

However, the creep resistance of 0.5Mn(LL) is still significantly improved compared with 0.5Mn(HL), confirming the promising and major role of fine and densely distributed dispersoids on the creep properties. In general, if the elevated-temperature properties are important, then 1Mn(LL) with the new processing route (low-temperature homogenization in combination with low-temperature extrusion) is considered the best candidate because it shows the highest YS at 300 °C and the best creep resistance owing to its higher Mn content and the highest amount of fine dispersoids. On the other hand, 0.5Mn(LL), under the same new processing route, has the highest YS at room temperature after T6 aging, and its YS at 300 °C is the second-highest among all the conditions (Figure 4 and Figure 6). Although 0.5Mn(LL) has an intermediate creep resistance, it provides a better compromise between the room-temperature properties after T6 and elevated-temperature properties after thermal exposure.

### 3.5. Modified Processing Route for Al-Mg-Si-Mn Alloys

This investigation has demonstrated that the application of a low-temperature homogenization treatment (400 °C) can result in the retention of a high density of fine α-Al(FeMn)Si dispersoids, which can increase the strength at room and elevated temperatures and improve the creep resistance. Commercially, AA6082 extrusion billets are typically homogenized above the Mg_2_Si solvus temperature to: (1) remove low melting point phases, (2) reduce microsegregation, (3) transform β-Al-Fe-Si intermetallics to α-Al-Fe-Si intermetallics, and (4) allow Mg_2_Si to be re-precipitated in a fine form. Although not widely studied, it has also been demonstrated that considerable coarsening and spheroidization of Fe/Mn-bearing dispersoids can occur during extrusion because of the enhanced diffusion rate during hot deformation. Reducing the extrusion temperature to 350 °C significantly reduced this effect. In practice, AA6082 is not often extruded with a billet temperature of less than 450 °C to ensure that the extrusion pressure is within the commercial press and extrusion die capabilities, and to allow Mg_2_Si to be readily dissolved during extrusion. Therefore, the proposed new processing route with a combination of low-temperature homogenization and extrusion is a departure from the conventional route, but the potential to enhance room/elevated-temperature mechanical properties and creep resistance for specific applications is promising and worthy of further exploration.

## 4. Conclusions

- Compared to coarse α-Al(FeMn)Si dispersoids formed during high-temperature homogenization, fine and densely distributed dispersoids resulting from low-temperature homogenization followed by low- and high-temperature extrusion remarkably improved the room-temperature yield strength of Al-Mg-Si-Mn alloys in T5/T6 states by 72–77 MPa (≈28%).
- α-Al(FeMn)Si dispersoids formed during low-temperature homogenization experienced less coarsening when the alloy was subsequently extruded at a low temperature of 350 °C compared to a more conventional high-temperature extrusion of 500 °C, resulting in higher yield strengths at both room and elevated temperatures.
- The yield strengths at 300 °C after thermal exposure at 300 °C/100 h were largely controlled by the size and distribution of α-Al(FeMn)Si dispersoids because of the instability of β″ precipitates at this temperature. Compared to the base case with coarse dispersoids, fine and densely distributed dispersoids were generated with the new processing route in both 0.5% Mn and 1% Mn alloys. Low homogenization and extrusion temperatures more than doubled the yield strength at 300 °C. In addition, finer dispersoids maintained by extrusion at 350 °C improved the yield strength at 300 °C by 17% compared to the extrusions at 500 °C.
- The creep at elevated temperature was controlled by a dislocation climb mechanism with a true stress exponent of 4.2–4.5. The creep resistance was greatly improved by an order of magnitude from the coarse dispersoid condition to one with fine and densely distributed dispersoids resulting from the new processing route.
- The 1% Mn alloy with the new processing route utilizing low homogenization and extrusion temperatures demonstrated the highest yield strength at 300 °C and the best creep resistance among all the conditions studied, while the lower Mn variant (0.5% Mn) provided a better compromise between the T6 room-temperature properties and the elevated-temperature properties after thermal exposure.

## Figures and Tables

**Table 1:** Heat treatments applied to the two alloys.

Alloys	Homogenization Parameter	Extrusion Temperature	Solution Heat Treatment Prior to Aging	Alloy Codes
0.5% Mn	550 °C/5 h	350 °C	540 °C/10 min (T6)	0.5Mn(HL)
	400 °C/5 h	500 °C	Not applied (T5)	0.5Mn(LH) [8]
		350 °C	540 °C/10 min (T6)	0.5Mn(LL)
1% Mn	400 °C/5 h	500 °C	Not applied (T5)	1Mn(LH) [8]
		350 °C	540 °C/10 min (T6)	1Mn(LL)

Note: T5 included quenching the extruded rods in a water bath as they exited the press, which was followed by aging at 180 °C for 5 h [8].

**Table 2:** Quantitative TEM results of dispersoids in experimental alloys.

Alloys	As-Extruded State			Solutionized State		
	$\bar{D}$	$N_{v},\;\;µm^{-3}$	$f_{d},\%$	$\bar{D}$	$N_{v},\;\;µm^{-3}$	$f_{d},\%$
0.5Mn(HL)	147 (±48.0)	5.3 (±2.5)	0.69 (±0.3)	138.3 (±54)	5.6 (±2.5)	0.71 (±0.3)
0.5Mn(LL)	25 (±1.7)	859 (±65)	0.62 (±0.1)	33 (±3.4)	387 (±51)	0.67 (±0.1)
1Mn(LL)	25 (±1.8)	1552 (±121)	0.86 (±0.1)	32 (±2.6)	588 (±55)	0.87 (±0.1)

**Table 3:** Summary of quantitative TEM results of dispersoids and YS under various conditions.

Alloy Code	Dispersoids			YS (MPa)		Reference
	*d*, nm	*N_v_*, µm^−3^	*f_d_*, %	RT	300 °C	
After T6/T5						
0.5Mn(HL)	138.3 (±54)	5.6 (±2.5)	0.71 (±0.3)	263		In this study
0.5Mn(LH)	40.3 (±2.5)	316.5 (±46)	0.64 (±0.1)	335		[8]
0.5Mn(LL)	33 (±3.4)	387 (±51)	0.67 (±0.1)	340		In this study
1Mn(LH)	40.2 (±2.5)	418.3 (±34)	0.93 (±0.1)	288		[8]
1Mn(LL)	32 (±2.6)	588 (±55)	0.87 (±0.1)	305		In this study
After thermal exposure at 300 °C/100 h						
0.5Mn(HL)	137.6 (±48.0)	5.9 (±2.5)	0.71 (±0.3)	60	30	In this study
0.5Mn(LH)	38.1 (±2.0)	353.9 (±62)	0.64 (±0.2)	99	52	[8]
0.5Mn(LL)	32 (±2.7)	391 (±61)	0.67 (±0.1)	119	61	In this study
1Mn(LH)	43.1 (±2.7)	418.3 (±41)	0.94 (±0.14)	106	58	[8]
1Mn(LL)	32 (±3.1)	594 (±65)	0.87 (±0.1)	134	68	In this study

## Data Availability

The raw/processed data required to reproduce these findings cannot be shared at this time as the data also forms part of an ongoing study.
